# Inhibition of EphA3 Expression in Tumour Stromal Cells Suppresses Tumour Growth and Progression

**DOI:** 10.3390/cancers15184646

**Published:** 2023-09-20

**Authors:** Mary E. Vail, Rae H. Farnsworth, Linda Hii, Stacey Allen, Sakshi Arora, Robin L. Anderson, Ross A. Dickins, Akira Orimo, Sunny Z. Wu, Alexander Swarbrick, Andrew M. Scott, Peter W. Janes

**Affiliations:** 1 Olivia Newton-John Cancer Research Institute and School of Cancer Medicine, La Trobe University, Heidelberg, VIC 3084, Australia; 2 Biomedicine Discovery Institute and Department of Biochemistry and Molecular Biology, Monash University, Clayton, VIC 3800, Australia; 3 Australian Centre for Blood Diseases, Monash University, Melbourne, VIC 3004, Australia; 4 Department of Pathology and Oncology, School of Medicine, Juntendo University, Tokyo 113-8421, Japan; 5 Garvan Institute of Medical Research and School of Clinical Medicine, University of NSW, Darlinghurst, NSW 2010, Australia

**Keywords:** Eph receptor, tumour microenvironment, cancer-associated fibroblast, tumour stroma, tumour angiogenesis

## Abstract

**Simple Summary:**

We have identified the cell guidance receptor EphA3 on distinct stromal/fibroblast-like cell types in the tumour microenvironment (TME) that promote growth and angiogenesis, both in human cancers and in mouse tumour models. To investigate its role in the TME, we developed novel transgenic mice with inducible shRNA-mediated knockdown of EphA3 expression. Knockdown of EphA3 expression in the TME in syngeneic mouse tumours reduced mesenchymal stromal/fibroblast-like cells in tumours, with corresponding reductions in tumour vasculature and tumour growth, and increased immune infiltrate, indicating an important role in tumour progression.

**Abstract:**

Tumour progression relies on interactions with untransformed cells in the tumour microenvironment (TME), including cancer-associated fibroblasts (CAFs), which promote blood supply, tumour progression, and immune evasion. Eph receptor tyrosine kinases are cell guidance receptors that are most active during development but re-emerge in cancer and are recognised drug targets. EphA3 is overexpressed in a wide range of tumour types, and we previously found expression particularly in stromal and vascular tissues of the TME. To investigate its role in the TME, we generated transgenic mice with inducible shRNA-mediated knockdown of EphA3 expression. EphA3 knockdown was confirmed in aortic mesenchymal stem cells (MSCs), which displayed reduced angiogenic capacity. In mice with syngeneic lung tumours, EphA3 knockdown reduced vasculature and CAF/MSC-like cells in tumours, and inhibited tumour growth, which was confirmed also in a melanoma model. Single cell RNA sequencing analysis of multiple human tumour types confirmed EphA3 expression in CAFs, including in breast cancer, where EphA3 was particularly prominent in perivascular- and myofibroblast-like CAFs. Our results thus indicate expression of the cell guidance receptor EphA3 in distinct CAF subpopulations is important in supporting tumour angiogenesis and tumour growth, highlighting its potential as a therapeutic target.

## 1. Introduction

It is now widely recognised that tumour development depends not only on genetic alterations and ensuing protein expression changes in tumour cells, but also on complex interplay with the tumour microenvironment (TME). Thus, cells from surrounding tissues, or recruited from the bone marrow, contribute to the tumour stroma and vasculature, and provide structural and nutrient support that promotes tumour growth [1]. Reciprocal signalling crosstalk between tumour cells and the stroma and vasculature, as well as immune cell infiltrates, can further promote tumour cell survival, proliferation, and resistance to therapy [2,3,4]. Various cell types contributing to the TME include mesenchymal stromal cells (MSCs) and cancer-associated fibroblasts (CAFs), which share many features in common, as well as endothelial cells and myeloid cell types (myeloid-derived suppressor cells and tumour-associated macrophages) [5]. Together, these non-transformed cells promote tumour growth via neo-angiogenesis, immune evasion, metastasis and drug resistance [1,6,7]. Compared to genetically unstable tumour cells that can adapt to avoid targeted intervention, these cell types in the TME are genetically stable, and are thus of major interest as therapeutic targets.

Eph receptors make up the largest family of receptor tyrosine kinases (RTKs) and are distinguished from other RTKs by binding to cell-bound ligands (ephrins), interacting across cell-cell junctions and controlling cell adhesion and segregation, cell fate, and survival. They are expressed most prominently during development, where they control cell guidance and differentiation during diverse processes including tissue boundary formation, organogenesis, and development of the neural, vascular, and immune systems [8,9,10]. While generally expressed less abundantly in adults, elevated levels of Ephs commonly re-emerge in cancer, associated with tumour stem/progenitor cells, neo-angiogenesis, invasion, and metastasis [11,12,13]. As a result, they are attractive therapeutic targets [14]. Moreover, elevated Eph receptor expression has been detected in the tumour microenvironment, as well as in tumour cells [13,15,16,17], including during tumour neo-angiogenesis, as exemplified by the role of EphA2 in endothelial cell migration and tumour vessel assembly [18,19,20].

We previously found that EphA3, known to be overexpressed in haematopoietic malignancies and many solid tumour types [21], is most commonly expressed in the tumour microenvironment in human tumour samples, but not in normal tissues [22]. This was also evident in mouse xenograft tumours, where EphA3 positive cells were detectable in the stroma and vasculature, while the tumour cell lines themselves lacked EphA3 expression. EphA3-positive stromal cells were found to originate from the bone marrow, and when isolated and cultured, they exhibited properties characteristic of MSCs. Furthermore, treatment of mice with an antibody against EphA3 was sufficient to disrupt the stroma and vasculature and inhibit tumour growth [22]. Similarly, hypoxia-induced EphA3 expression was observed in perivascular MSCs from regenerating endometrium, supporting a role for EphA3 in hypoxia-driven neo-vascularisation [23]. Thus, we set out to better define the expression and function of EphA3 in the TME, and to determine its requirement for supporting tumour development, using syngeneic tumours in transgenic mice with inducible shRNA-mediated knockdown of EphA3 expression.

## 2. Materials and Methods

### 2.1. Cell Culture

Lewis lung carcinoma (LLC), B16F10 melanoma, and fibroblast cell lines were grown in DMEM/10% foetal calf serum. Primary mesenchymal stromal cells were isolated and maintained, as previously described [23], under hypoxia (2% O_2_) to maintain EphA3 expression. 5B3 ES cells were cultured on a mouse embryonic fibroblast (MEF) feeder layer as previously described [24].

### 2.2. shRNA Plasmid Construct and Viral Transduction

shRNA sequences targeting mouse EphA3 were designed using DSIR [25] and Sensor [26] prediction tools. Briefly, 97-mer microRNA-adapted short hairpin RNAs (shRNA-mirs) consisting of the target-specific shRNA embedded in a miR30 backbone were PCR amplified and subcloned into the LMP (MSCV-LTR-miR30-PGK-IRES-GFP) retroviral vector using EcoR1 and XhoI, as previously described [24]. Six EphA3-targeting shRNAs and two control shRNAs targeting Firefly or Renilla luciferases (Luc.1309 and Ren.713 respectively) were tested for knockdown efficacy in MEFs via retroviral transduction. Briefly, EphA3 or control shRNA LMP constructs were transfected into 293 Phoenix cells or co-transfected into 293T cells with pCLEco. After 2 days, the viral supernatant was recovered and used to transduce wild-type MEFs in 6-well plates. Viral supernatants were diluted in growth medium containing 8 µg/mL hexadimethrine bromide (polybrene, Sigma Aldrich, St. Louis, MO, USA), and cells were transduced via centrifugation at 1500× *g* for 1 h at 32 °C. Transduced cells were selected using puromycin resistance and analysed for GFP fluorescence and EphA3 expression via flow cytometry and quantitative RT-PCR.

### 2.3. Generation of EphA3 shRNA Mice

shRNA EphA3.3466 was inserted into the pColTGM (Col1A TREtight-eGFP-miR30) plasmid (EcoR1/XhoI restriction sites), containing a tetracycline (Tet)-responsive TRE-tight promoter element [27] driving expression of shRNA embedded in the miR30 structure, and a GFP reporter [28]. The modified Col1A locus was verified via sequencing across the insertion region. The pColTGM EphA3-3466 plasmid was introduced into 5B3 C57BL/6 mouse embryonic stem (ES) cells cultured on an established MEF feeder layer [24]. These ES cells contain an ‘frt-landing pad’ in the Col1a1 locus to enable targeted integration in the presence of Flp DNA recombinase, achieved by co-transfection with the pCAGs-Flpe plasmid [29]. Stable clones were selected using Hygromycin resistance, and colonies screened for construct integration by PCR. Positive clones were isolated and used for blastocyst injection, and impregnation of C57BL/6 mice. EphA3 shRNA-positive offspring determined via PCR genotyping [24] were bred to homozygosity, and crossed to C57BL/6 mice expressing the Tet-ON transactivator M2rtTA [28], driven by the widely expressed Rosa26 (R26) promoter [30,31], to produce EphA3 shRNA/R26rtTA double-homozygous mice. C57BL/6 mice that were double-homozygous for the control Renilla luciferase shRNA (Ren.713) and R26rtTA were similarly produced. All strains were born at expected Mendelian ratios and appeared healthy and fertile.

### 2.4. Quantitative RT-PCR (qRT-PCR)

RNA was extracted using Isolate II RNA kit (Bioline). Samples were analysed via SYBR Green qRT-PCR, using a Rotorgene 6000 (Corbett Research). Relative gene expression was determined using tubulin β4a as a reference gene.

qRT-PCR primers: EphA3 2721For CAAGTTCGAGCAGATCGTCA, 2879 Rev CCGTTAAGCCAATCACCAGT; tubβ4a For CCAGATCTTTCGGCCAGACAAC, Rev CCCTCGGTGTAGTGACCCTTG.

PCR primers for genotyping: 

EphA3 sh 3466For CCACAGATGTATTAAAAGAAAC, RBG Rev GAAAGAACAATCAAGGGTCC; Ren 713For ATGTATAGATAAGCATTATAATTCC, RBG Rev GAAAGAACAATCAAGGGTCC; rtTA For CGGTATCGAAGGCCTGACGACAAGG, Rev AGAAGCCTTGCTGACACAGGAACGC.

### 2.5. Syngeneic Tumour Models

Aged-matched controls and EphA3 shRNA mice (males, 6–14 weeks age) were fed on mouse food pellets containing Doxycycline (600 mg/kg, Specialty Feeds, Glen Forrest, WA, 6071, Australia) for 2 weeks prior to experiments. For syngeneic tumour models, we used LLC cells and B16F10 melanoma cells, which we previously characterized as EphA3-negative and EphA3-positive, respectively. 5 million LLC or 2 million B16F10 cells were injected subcutaneously in the flank of the control and EphA3 shRNA mice (*n* ≥ 5), and tumour growth was monitored by calipers until ethical endpoint or at earlier time points for analysis, as specified in the figure legends. The results show representative data from at least three replicate experiments. All animals were handled in strict accordance with good animal practice, as defined by the National Health and Medical Research Council (Australia) Code of Practice for the Care and Use of Animals for Experimental Purposes, and approved by the Austin Hospital and Monash University Animal Ethics Committees.

### 2.6. Aortic Ring Sprouting Assay

The aortic ring sprouting assay was performed as previously described [32]. In brief, thoracic aortae were dissected from 3–5 mice at 8–10 weeks of age, cleaned, and cut into rings approximately 5 mm thick. Between 15 and 20 rings per mouse were pooled, and a minimum of 40 rings were used. Rings were serum-starved overnight and embedded the next day in type I collagen (1 mg/mL), before incubation in Opti-MEM culture medium supplemented with 2.5% FBS and 30 ng/mL VEGF. After 7 days, rings were stained with Rhodamine-Lectin I (GSL1, Vector Laboratories, Newark, CA, USA) (0.1 mg/mL) to stain aortic sprouts, along with antibodies as listed in figure legends. Imaging was carried out with an inverted confocal microscope (Leica SP5 or Zeiss 980), using Z-stack maximum projections. For quantitation experiments, low-resolution 3 × 3 tile scan images were taken with a 10 × lens. Image processing was performed with software from Zeiss (Oberkochen, Germany) and Imaris (Oxford Instruments, Abingdon, UK).

### 2.7. Antibodies

NG2 rabbit polyclonal antibody (ab5329) was from Millipore; rat anti-mouse CD90-PB (30-H12), CD45-PB (30F-11) and Sca1-PE/Cy7 (D7) monoclonal antibodies were from BioLegend, San Diego, CA, USA; rat anti-mouse CD8 (4SM15) and TER119 (ef450 conjugated 48-5921-80) monoclonal antibodies were from eBioscience (ThermoFischer, San Diego, CA, USA); CD31 rat anti-mouse monoclonal antibody (Clone MEC 13.3) was from Becton Dickinson; rabbit transgelin (155272) polyclonal and alpha smooth muscle actin (SMA) (ab124964) monoclonal antibodies were from Abcam (Cambridge, UK); α-EphA3 mouse monoclonal IIIA4 and sheep polyclonal antibodies have been previously described [22,33]. Alexa Fluor-labelled secondary anti-rabbit and anti-sheep antibodies were from Invitrogen (ThermoFischer, San Diego, CA, USA).

### 2.8. Tissue Immunohistochemistry (IHC) and Immunofluorescence (IF)

Mouse tumours were embedded in optimal cutting temperature (OCT) medium. When relevant, to preserve GFP, tumours were fixed in 4% PFA overnight, and transferred to 30% sucrose for 24 h prior to embedding and freezing in OCT. Formalin-fixed, paraffin-embedded human tumour samples were sectioned and stained with the primary antibodies listed above. For the IHC antibody, binding was detected using the IHC Vector Labs ABC staining kit, before haemotoxylin counterstaining. Stained sections were scanned using the Aperio Scanscope XT (Leica Biosystems, Wetzlar, Germany), and biomarker analysis was conducted using the Aperio image analysis Positive Pixel Count v9 algorithm. For immunofluorescence staining of human tumours, Alexa Fluor-labelled secondary antibodies (Invitrogen, Waltham, MA, USA) were used, nuclei were stained with DAPI, and imaging was carried out using a Zeiss LSM 980 confocal microscope (displayed as a maximum projection of a 6 micron z-stack), using Zeiss imaging software. Human tissue samples were collected following approval from the Austin Health Human Ethics Committee.

### 2.9. Flow Cytometry

Cells stained with the indicated primary antibodies and Alexa Fluor-conjugated secondary antibodies were analysed via flow cytometry using LSRII and FACS Canto II flow cytometers (Becton Dickinson). Dead cells detected with propidium iodide were excluded, and FLOWJO software version 10.5.3 (TreeStar, San Jose, CA, USA) was used for raw data analysis and multivariate compensation.

### 2.10. Statistical Analysis

Graphs show individual data points, with mean and standard error, and *p* value range to indicate statistical significance, as determined by unpaired two-tailed student’s *t* test, or as otherwise specified.

## 3. Results

### 3.1. EphA3 Is Expressed on MSC-like Cells Associated with Angiogenesis

**Figure 1:**
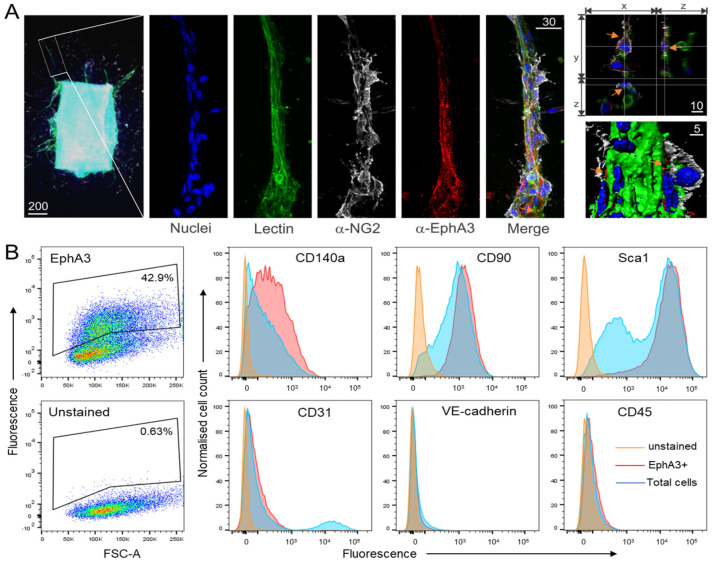
EphA3 is expressed on cells associated with angiogenesis. (**A**) Aortic explants from mice were incubated in Matrigel and VEGF-A to stimulate vessel sprouting, and then stained with Rhodamine-lectin (marking vessels), DAPI (marking nuclei), and antibodies for EphA3 and NG2 (perivascular cell marker). Images were obtained using confocal fluorescence microscopy. A vessel sprout indicated in the low-magnification image of a sprouting aortic explant (left panel) is shown at a higher magnification (middle panels) using combined z-stack images of individual and merged channels, as indicated. Right panels: the top panel shows a single xy and z-stack series of a region showing EphA3 staining (arrows) in NG2-positive perivascular cells. White lines in images indicate planes of associated xy, xz and yz sections; the bottom panel shows three-dimensional rendering of a z-stack showing EphA3 staining at boundaries between perivascular cells and lectin-stained endothelial cells (arrows). Scale bars in microns. (**B**) Primary aortic cell cultures were stained with antibodies against EphA3 (Alexa-647 conjugated), and markers for MSCs (CD140a, CD90, Sca1), endothelial cells (CD31, VE-cadherin), and haematopoietic cells (CD45) before analysis by flow cytometry. Dot plots (left) show EphA3 staining (fluorescence versus forward scatter) compared to unstained control cells; histograms show co-staining of EphA3 positive or total viable cells, with the indicated markers or unstained cells as control.

We first investigated EphA3 expression in MSC-supported angiogenesis using the aortic ring model of sprouting angiogenesis, where a small piece of mouse aorta explant is embedded in type I collagen and incubated in the presence of vascular endothelial growth factor-A (VEGF-A) to encourage vessel sprouting [32]. After 1 week, explants were stained with fluorescent lectin (to mark endothelial sprouts) [32], and with antibodies against EphA3 and NG2, a marker expressed on pericytes and other perivascular MSC-like cells that reside in the outer wall of blood vessels and maintain vessel integrity [34]. EphA3 expression was clearly detected on new sprouts, and was evident in NG2+ cells, particularly at boundaries with lectin-stained sprouts (Figure 1A). We also used flow cytometry to analyse the primary cells extracted from mice aortae and grown in tissue culture. EphA3 expression was detected in a significant population of cells, which were found to preferentially co-express MSC markers such as CD140a (PDGFRα), CD90 and Sca1, consistent with EphA3 expression in vessel-associated MSCs (Figure 1B). In comparison, the cultured EphA3+ cells expressed lower levels of cell surface markers associated with endothelial cells (CD31, VE-cadherin), or with hematopoietic cells (CD45).

### 3.2. Generation of Mice with Inducible shRNA-Mediated Knockdown of EphA3

To investigate if EphA3 functionally supports neo-angiogenesis, we developed shRNA transgenic mice, allowing doxycycline-induced expression of shRNA, linked with green fluorescent protein (GFP) as a reporter [24,35]. This approach was chosen to avoid the mortality associated with germline knockout [36], and the possible compensatory expression of other redundant Ephs in surviving viable mice. Induced knockdown also mimics partial systemic reduction associated with therapeutic inhibition. Candidate EphA3-specific shRNA sequences (Table S1) were screened for inhibition of EphA3 expression in mouse embryonic fibroblasts (MEFs), which robustly express EphA3, compared with a control shRNA targeting the Renilla luciferase gene (Ren.713) (Figure S1). An optimal EphA3 shRNA (3466), with no significant homology to other Eph family genes and a control shRNA (Ren.713) were placed in a tetracycline (Tet)-inducible expression system with a GFP reporter, and used to generate transgenic mice also co-expressing the Tet-ON transactivator M2rtTA, driven by the widely expressed Rosa26 (R26) promoter [30,31] (see Section 2 for more details).

**Figure 2:**
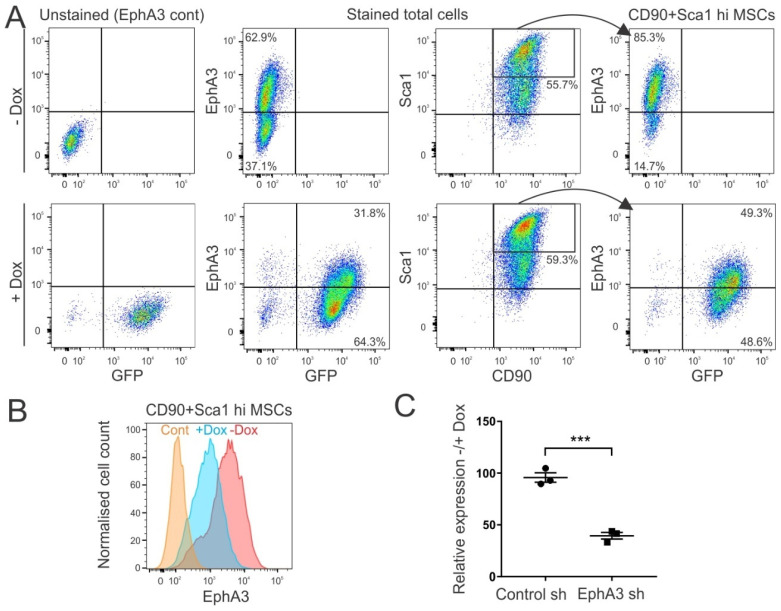
Decreased EphA3 expression in aortic MSCs derived from EphA3 shRNA mice. Aortic cells derived from control and EphA3 shRNA mice were cultured with or without doxycycline (Dox) to induce shRNA and GFP expression, and analysed via FACS for expression of MSC markers (CD90, Sca1), EphA3, and GFP (shRNA reporter). (**A**) Representative FACS plots of aortic MSCs from EphA3 shRNA mice with or without doxycycline, showing total live cells, or just MSCs (CD90+/Sca1 high, right panels), stained with antibodies against EphA3, versus shRNA/GFP expression. Left panels show cells unstained for EphA3 as controls. (**B**) Overlaid histograms of EphA3 staining of CD90+/Sca1-high MSC populations from EphA3 shRNA mice, with and without doxycycline, with unstained cells as control (Cont). (**C**) Graph summarising the relative EphA3 staining (ratio of geometric mean fluorescence) of three aortic MSC lines, each from control versus EphA3 shRNA (sh) mice, cultured with or without doxycycline. *** *p* < 0.001.

To test and quantify inducible EphA3 knockdown in these mice, we again isolated primary cells from the aortae of EphA3 shRNA or control shRNA mice, and cultured them in vitro with or without the tetracycline analogue doxycycline. FACS analysis of total cell populations showed robust induction of GFP expression upon doxycycline stimulation, accompanied by a marked decrease in EphA3 expression, compared to aortic cell cultures from control shRNA mice (Figure 2A). As above, EphA3 expression was particularly prominent in MSC-like cells expressing CD90 and high levels of Sca1 (Figure S2), and expression was decreased by approximately 70% upon doxycycline induction of EphA3 shRNA, compared to controls (Figure 2B,C). 

### 3.3. Reduced Angiogenic Sprouting of Aortic Explants from EphA3 shRNA Mice

**Figure 3:**
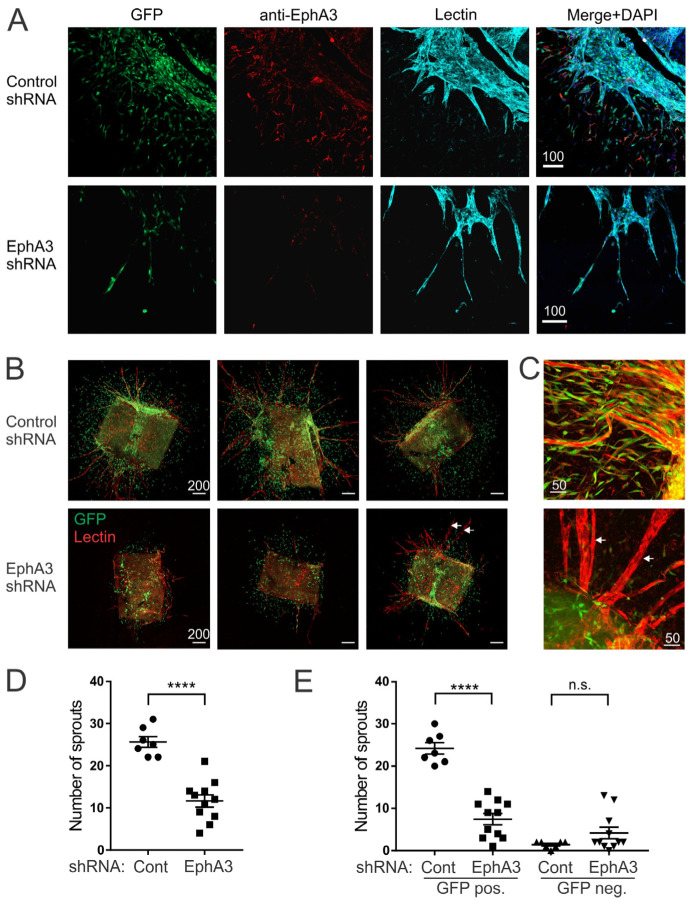
Reduced angiogenic sprouting of aortic explants from EphA3 shRNA mice. Multiple aortic slices from control (Cont) or EphA3 shRNA mice (*n* = 4 mice/group) were grown in collagen under angiogenic conditions with doxycycline to induce shRNA expression. (**A**) Fluorescence microscopy of newly formed vessel sprouts from control or EphA3 shRNA mice, marked by lectin (cyan) and anti-EphA3 antibody staining (red). GFP expression (green) marks cells expressing shRNA, and nuclei are stained with DAPI (dark blue). (**B**) Low-magnification imaging of whole explants to show overall sprouting, with rhodamine-lectin in red, and higher-magnification images in (**C**). Arrows indicate occasional sprouts, typically not associated with GFP+ cells, arising in EphA3 shRNA aortic rings. (**D**,**E**) Quantitation of sprout number from aortic explants (from four individual mice per group). Graphs show total sprouts (**D**), or sprouts grouped by association with GFP (shRNA) expression (**E**), showing mean and SEM. **** *p* < 0.0001, n.s. not significant.

As a functional readout in vitro, we compared the angiogenic sprouting of aortic rings recovered from EphA3 and control shRNA mice. These were cultured in the presence of doxycycline to induce shRNA-GFP expression, and stained with fluorescently labelled lectin to mark vessel sprouts. Immunofluorescence staining confirmed expression of EphA3 in cells with a mesenchymal, migratory appearance, around lectin-stained sprouts, which was notably reduced in explants from EphA3 shRNA mice (Figure 3A). Imaging of whole explants also showed a clear reduction in sprouting in aortic explants from EphA3 shRNA mice compared to control mice (Figure 3B,D), and the few sprouts that did form in EphA3 shRNA explants had a less defined structure and less associated shRNA/GFP+ cells, indicating that EphA3 knockdown in these cells is detrimental to vessel formation (Figure 3B,C). Accordingly, reduced vessel formation was dependent on shRNA/GFP expression (Figure 3E). These data indicate that EphA3 expression in MSCs plays a supportive role in angiogenesis, likely governing association of perivascular MSC-like cells within new vessel sprouts.

### 3.4. Bone Marrow-Derived Cells from EphA3 Knockdown Mice Show Reduced Colony-Forming Potential

**Figure 4:**
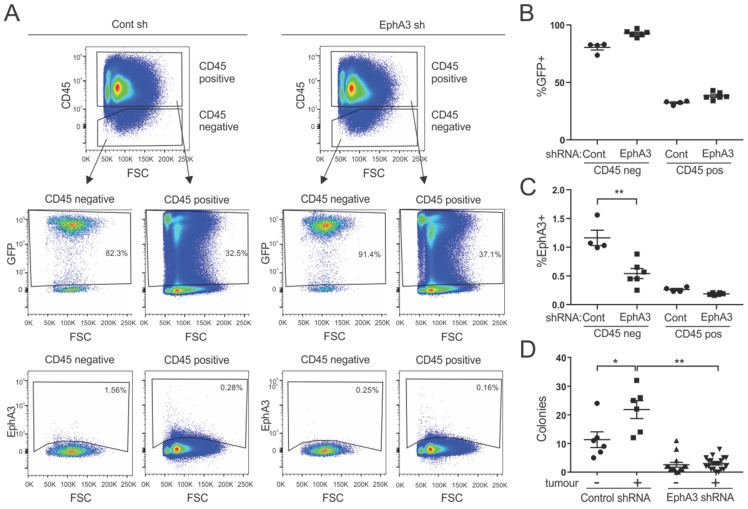
Analysis of bone marrow cells from control and EphA3 shRNA mice bearing subcutaneous LLC tumours. (**A**–**C**) Control (Cont) and EphA3 shRNA mice fed on doxycycline were injected subcutaneously with LLC tumour cells, and after 6 days, bone marrow was recovered and stained with antibodies against CD45 (hematopoietic marker), TER119 (erythroid marker), and EphA3, prior to analysis by flow cytometry. (**A**) Representative flow cytometry profiles of TER119 negative bone marrow cells showing GFP (shRNA) and EphA3 expression in CD45-negative and -positive cells. (**B**,**C**) Graphs summarising mean % staining from multiple mice (+/– SEM, *n* = 4–6, ** *p* < 0.01). (**D**) Equal numbers of unsorted bone marrow cells flushed from femurs of shRNA mice with or without LLC tumours were grown in 6-well plates, and after 17 days, colonies were stained with crystal violet. The graph shows the colony number per 15 million cells extracted from individual mice, with mean and standard error (** *p* < 0.01, * *p* < 0.05).

To investigate a functional role for EphA3 in cells contributing to the tumour microenvironment, we used a Lewis lung carcinoma (LLC) syngeneic mouse tumour model, chosen because we showed previously that EphA3 was expressed in the TME, including MSCs recruited from the bone marrow, but not in the tumour cells [22]. Mice were fed doxycycline-containing chow for two weeks before tumour cell injection, and thereafter to retain shRNA expression during tumour growth. As we previously detected elevated EphA3 expression in bone marrow-derived MSCs in human tumour xenograft models [22], we analysed bone marrow cells from EphA3 and control shRNA hosts bearing LLC tumours. Hematopoietic (CD45+) and non-hematopoietic (CD45−) cells were analysed for shRNA (GFP) and EphA3 expression. This showed comparable shRNA/GFP expression in bone marrow cells from both EphA3 and control shRNA mice, with GFP expression in approximately 80% of CD45 negative cells (Figure 4A,B). Interestingly, GFP expression in CD45-positive cells from these mice was substantially lower, detectable in around 30% of cells, possibly owing to the limited activity of the rtTA trans-activator in B and T cells [37]. EphA3 was expressed in a greater proportion of non-hematopoietic bone marrow cells compared to CD45-positive cells, and this expression was significantly reduced in bone marrow from mice with EphA3 shRNA, compared to control shRNA (Figure 4C). We then analysed bone marrow cells extracted from these mice for their ability to form colonies on plastic tissue culture plates, which is a characteristic feature of multipotent MSCs. Bone marrow cells from control mice with tumours exhibited markedly increased colony-forming ability, compared to mice without tumours, reflecting the upregulation of stem cell content due to tumour burden [38]. In comparison, colony formation potential was clearly abrogated in bone marrow samples from mice with EphA3 knockdown (Figure 4D), suggesting a role for EphA3 in MSC survival and replicative capacity.

### 3.5. Tumours from EphA3 Knockdown Mice Exhibit an Altered TME and Reduced Tumour Growth

**Figure 5:**
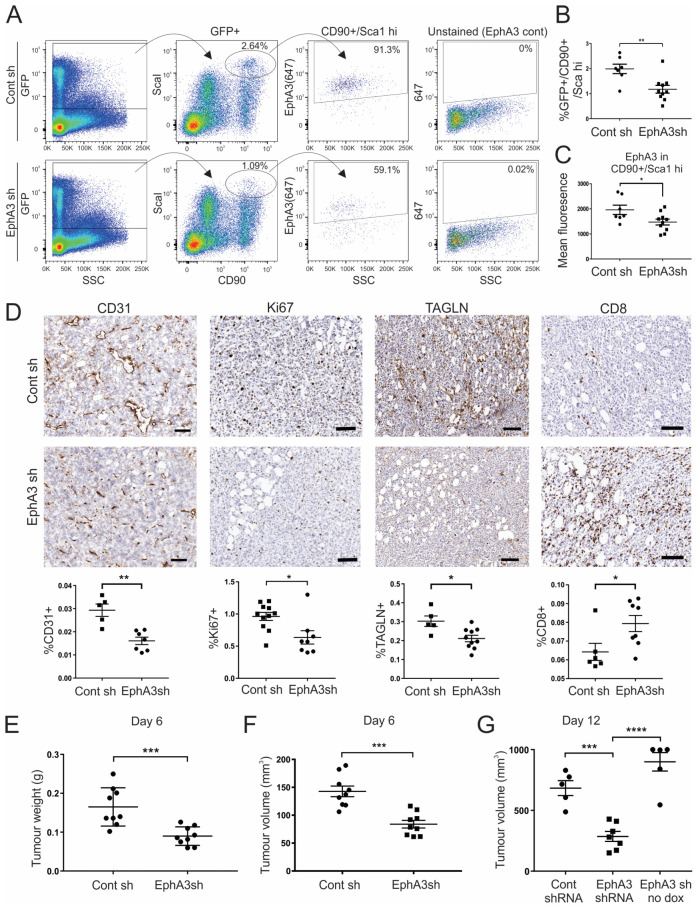
Effects of doxycycline-induced EphA3 knockdown in mice on LLC tumour allografts. (**A**–**C**) Flow cytometry analysis of day 6 subcutaneous tumours. (**A**) Representative plots show GFP (shRNA)-expressing cells stained for MSC markers CD90 and Sca1, and EphA3 staining in CD90+/Sca1-high cells. (**B**) Comparison of %CD90+/Sca1-high cells in tumours from EphA3 versus control (Cont) shRNA (sh) mice. (**C**) EphA3 staining in GFP+/CD90+/Sca1-high MSCs. (**D**) Immunohistochemistry of tumours stained for CD31+ endothelial cells, Ki67 (proliferation), Transgelin (TAGLN)+ fibroblasts, and CD8+ T cells (scale bars 100 µm). The graphs below show quantitation analysis of whole sections from individual tumours. (**E**,**F**) Comparison of tumour weight (**E**) and volume (**F**) at day 6. (**G**) Tumour volumes at day 12 in control and EphA3 shRNA mice on doxycycline, or EphA3 shRNA mice without doxycycline (no dox). Graphs show mean +/– SEM. * *p* < 0.05, ** *p* < 0.01, *** *p* < 0.001, **** *p* < 0.0001.

We next compared LLC primary tumours grown subcutaneously in mice with doxycycline-induced shRNA expression. Analysis of early-stage tumours showed EphA3 was almost exclusively expressed in GFP+, CD90+/Sca1-high MSCs (Figure S3), and this population was significantly reduced in tumours from EphA3 shRNA host mice compared to control hosts (Figure 5A,B), suggesting reduced recruitment from the bone marrow. The remaining MSCs also had an overall decrease in EphA3 staining in EphA3 shRNA mice (Figure 5C and Figure S3). IHC analysis of tumours showed that those from EphA3 shRNA mice displayed significantly less staining of the endothelial marker CD31, indicative of reduced tumour angiogenesis, with a concomitant decrease in proliferation, as determined by Ki67 staining (Figure 5D). Since CAFs share many characteristics with MSCs, from which they can be derived [39], we also tested expression of the myofibroblast-expressed actin-binding protein Transgelin [40], a CAF marker known to be associated with angiogenesis and poor prognosis [41,42] (Figure 5D). Transgelin staining was decreased upon EphA3 knockdown, consistent with EphA3 marking pro-angiogenic fibroblasts in the TME. Multiplex immunofluorescence staining also showed EphA3 expression was associated with NG2+ cells, characteristic of perivascular fibroblasts, and this was also reduced in tumours from EphA3 shRNA mice (Figure S4). Interestingly, we also detected an increase in CD8+ T cells in tumours (Figure 5D and Figure S4), suggesting a possible role for EphA3-expressing cells in immune suppression, as reported for subsets of CAFs [40], although this was more variable, perhaps reflecting the simultaneous reduction of vessel formation which might restrict T cell infiltration. Notably, tumours in mice with doxycycline-induced EphA3 knockdown were significantly smaller/delayed (Figure 5E,F, and Figure S5), suggesting a functional role for EphA3 in MSCs/CAFs that promote tumour growth. We further confirmed a significant decrease in tumour growth over a longer timeframe in doxycycline-treated compared to untreated EphA3 shRNA mice, to rule out possible variation in transgenic mouse strains (Figure 5G). 

To analyse the effects of EphA3 knockdown in the TME of tumours in which the tumour cells also express EphA3, we used subcutaneous tumours of B16F10 mouse melanoma cells, which have robust EphA3 expression. Once again, EphA3 knockdown in mice significantly inhibited tumour growth compared to control knockdown mice (Figure S6), also indicating a role for EphA3 in the TME of tumours in which EphA3 is expressed in tumour cells. This is also supported by our analysis of available single-cell RNA sequence data from B16F10 tumours [43] (https://melanoma.cellgeni.sanger.ac.uk, accessed on 7 September 2023), showing EphA3 expression in CAFs (Figure S7A,B). 

### 3.6. EphA3 Is Expressed in Distinct CAF Subtypes in Human Tumours

**Figure 6:**
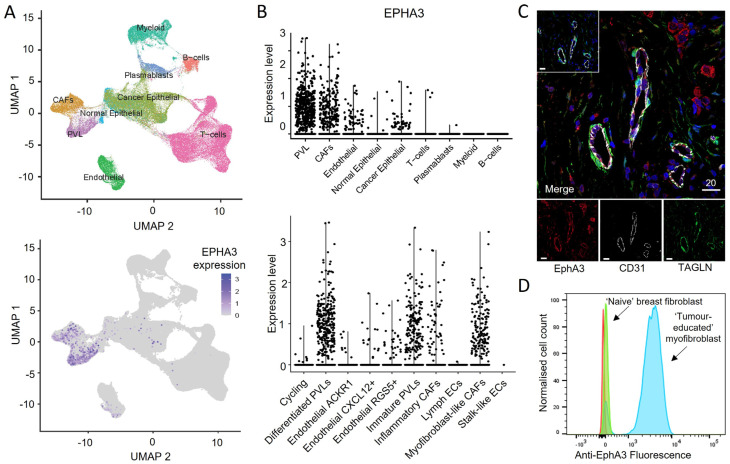
Identification of EphA3 expression in human breast cancer stromal cell subsets. Single-cell RNA sequencing of 130,246 cells from 26 early breast cancers was used to classify tumour and stromal cell subpopulations, which were analysed for EphA3 expression. (**A**) UMAP plots showing identified cell type clusters (top) and EphA3 expression (bottom). (**B**) Graphs depicting expression in broad cell subtypes (top), and in more defined stromal subsets (bottom, 20,148 cells). EphA3 is most prominent in cancer-associated fibroblasts (CAFs) and perivascular-like cells (PVL). ECs, endothelial cells. (**C**) Immunofluorescence staining of human breast tumour, imaged by confocal microscopy, showing staining of EphA3, CD31 and Transgelin (TAGLN), with individual channels below (inset: stained with anti-EphA3 antibody pre-blocked with recombinant EphA3 competition to verify specific binding). Scale bars indicate 20 microns. (**D**) Flow cytometry analysis of tumour-educated (blue) and naïve (green) human breast fibroblasts [47] (red = unstained).

Finally, we sought to characterise EphA3 expression in the TME of human tumours. We used a single-cell RNA sequencing analysis of 26 early-stage breast tumours [44] to analyse EphA3 expression in distinct cell types based on gene expression profiles (Figure 6). EphA3 was most prominent in cells expressing genes characteristic of CAFs and perivascular-like cells (PVLs), which also share MSC markers (Figure 6A,B). PVLs included more mature (smooth muscle cell-like) and immature (pericyte-like) cells. Interestingly, EphA3 was expressed in myofibroblasts and other immune-modulatory CAF subtypes [40] (Figure 6B), consistent with the altered T cell infiltrate in tumours in our mice following EphA3 knockdown. Markers co-expressed in EphA3+ cells included smooth-muscle actin (SMA, or ACTA2), Transgelin (TAGLN), and others associated with acto-myosin regulation (MYL9, MYH11, CALD1), TGFβ signalling (EGR1, CAV1), and pericytes (RGS5). Immunofluorescence staining of breast tumour tissue with antibodies against EphA3, Transgelin and the endothelial cell marker CD31 confirmed coincident expression at the protein level (Figure 6C), with some EphA3 staining also in Transgelin-negative cells, including endothelial cells, which is consistent with the RNA expression data. Our analysis of publicly available expression databases confirmed EphA3 expression in CAFs also from other human tumours, including melanoma [45] https://singlecell.broadinstitute.org/single_cell, (Figure S7C), and colon cancer [46] http://crcleukocyte.cancer-pku.cn/ (accessed on 7 September 2023).

Interestingly, we also tested EphA3 expression in a human breast fibroblast cell line that had been ‘tumour-educated’ by co-implantation with human breast tumour cells in a mouse xenograft model, inducing a myofibroblast-like CAF phenotype [47]. Flow cytometry analysis showed clearly upregulated expression of EphA3 compared to minimal expression on the control ‘tumour-naïve’ cell line, consistent with preferential expression on tumour-associated fibroblasts (Figure 6D). The mechanism of EphA3 upregulation in CAFs and their pro-tumorigenic role warrants further investigation.

## 4. Discussion

The tumour microenvironment is of increasing interest for therapeutic intervention, with anti-angiogenic and immune modulatory agents already used in the clinic. Eph receptors have long been recognised as potential therapeutic targets due to their deregulated expression in tumour cells. More recently, their detection in cells of the TME suggests that they may act more broadly in tumour development. We determined previously that EphA3 was frequently expressed in the stroma in a range of different human tumours, and in tumour cell xenografts in mice, even when not expressed in the tumour cells [22]. Bone marrow transplant experiments indicated that EphA3+ cells were predominately derived from the host bone marrow and, when isolated and propagated in culture, these cells exhibited features characteristic of mesenchymal stromal cells (MSCs), including multi-potency and expression of key MSC markers [22]. We also showed that treatment of EphA3+ MSCs with the agonistic EphA3 antibody IIIA4 caused cytoskeletal collapse, reducing adhesion and cell viability in vitro, and prolonged treatment of mice bearing tumours resulted in disruption of the tumour stroma and decreased tumour growth. These results indicated a functional role for these EphA3+ MSCs in tumour growth, which we have now further investigated.

Here, we have developed novel transgenic mice in which EphA3 expression can be specifically and temporally suppressed by shRNA interference in adult mice prior to tumour inoculation, thus avoiding the developmental and possible compensatory effects of germline gene knockout. Using syngeneic mouse tumour allografts of EphA3-negative LLC cells implanted in these mice, we show that EphA3 expression is detectable in the MSC/CAF-like cells of the tumour microenvironment, and that knockdown of EphA3 expression indeed has impacts on tumour growth. EphA3 suppression reduced levels of MSCs in tumours, including cells resembling perivascular and myofibroblast-like CAFs, and was associated with decreased blood vessel density and cell proliferation in tumours. Accordingly, EphA3 knockdown decreased vessel sprouting in tissue explants from mice, suggesting an important role for EphA3+ MSCs in supporting angiogenesis, as previously described for MSCs more generally [39]. Bone marrow-derived MSCs from these mice also showed significantly attenuated stem cell-like capacity, as measured by survival and reproduction in colony forming assays, consistent with the known roles of Ephs including EphA3 in supporting less differentiated, stem cell-like cells in tumours [12,48]. These data, combined with our previous work [22], are consistent with the bone marrow being a significant source of EphA3+ MSC/CAF-like cells in tumours, and points to a possible role for EphA3 in supporting their recruitment to tumours.

Other Eph receptors have previously been shown to be important for both normal and oncogenic angiogenesis, particularly EphB receptors and EphA2, along with their cognate ephrin ligands (ephrin-B1–3, ephrin-A1) [13,18,20]. EphA2 and ephrin-A1 are expressed in tumours and associated vessels, with mouse models indicating that EphA2 expression in vessels is important for efficient angiogenesis [19]. EphB4–ephrin-B2 interaction between endothelial cells and supporting perivascular cells (pericytes or smooth muscle cells) is also critical for angiogenesis [49,50]. Our results extend these observations, and indicate that EphA3 also has an important role in the TME on mesenchymal progenitor/fibroblast-like cells in tumours, which likely contribute to the perivascular niche and support angiogenesis. We provide mechanistic evidence for this using an in vitro angiogenic assay in which EphA3 knockdown reduced the numbers of MSCs interacting with aortic sprouts, and coincided with significantly reduced sprouting efficiency under hypoxic conditions. Indeed, MSCs are known to enhance tumour angiogenesis in response to hypoxic signalling, in a process analogous to wound-healing [51], and EphA3 is up-regulated by hypoxia, including during neo-angiogenesis [23,52]. 

Importantly, we also confirm EphA3 expression in similar cell subtypes in the TME of human cancers, including breast, colon and melanoma, by analysis of single-cell RNA sequencing data. Our data from early-stage breast tumours [44] shows that EphA3 is particularly expressed in stromal CAF populations that express genes typical of myofibroblast-like perivascular/smooth muscle cells, including SMA, Transgelin, and other genes associated with acto-myosin regulation. These CAFs also expressed genes associated with TGFβ signalling, known to drive a more aggressive mesenchymal tumour phenotype. Interestingly, myofibroblast-like and other CAF sub-populations expressing EphA3 are known to have immune-suppressive activity in tumours [53,54], and we observed increased cytotoxic T cell infiltration into tumours in mice with EphA3 knockdown, suggesting possible implications for enhanced anti-tumour immune response as a subject for future investigation. Our results therefore indicate an important role of EphA3 in the tumour microenvironment, and suggest the potential therapeutic utility of targeting EphA3-expressing CAFs in solid tumours. Interestingly, clinical studies testing the humanized anti-EphA3 antibody Ifabotuzumab (KB004) showed clinical activity particularly in patients with fibrotic myeloid diseases, supporting its targeting of the stromal microenvironment [55].

## 5. Conclusions

In conclusion, we identify the cell guidance receptor EphA3 as a novel marker in distinct myofibroblast-like perivascular/smooth muscle cells in the microenvironment of human tumours and in analogous mesenchymal/fibroblast cells in mouse tumour models. Our novel transgenic mice, with specific knockdown of EphA3 expression in the TME of implanted syngeneic tumours, showed reduced tumour growth and angiogenesis, and associated defects in stem-like and angiogenic properties in mesenchymal stromal progenitor cells. Our results thus indicate an important functional role of EphA3 in mesenchymal CAF-like cells recruited to tumours that promote angiogenesis and tumour growth, and suggest the potential of EphA3 as a therapeutic target in the TME of solid tumours.

## Data Availability

All data that support the findings of this study are available from the corresponding authors upon reasonable request.

## Supplementary Materials

The following supporting information can be downloaded at: https://www.mdpi.com/article/10.3390/cancers15184646/s1; Table S1. EphA3 shRNA sequences. Figure S1. Screening for EphA3 shRNA knockdown in mouse embryonic fibroblasts. Figure S2. EphA3 expression in aortic cell cultures distinguished by Sca1 expression. Figure S3. EphA3 expression in LLC tumour cell isolates distinguished by expression of CD90/Sca1. Figure S4. Immunofluorescence microscopy of LLC tumours from control and EphA3 shRNA mice. Figure S5. Tumour growth and survival of shRNA mice bearing LLC tumours. Figure S6. Effect of EphA3 knockdown on growth of B16F10 melanoma tumours. Figure S7. Analysis of single cell RNA sequence data of mouse and human melanomas shows EphA3 in CAF subtypes.

## References

[1] Quail D.F., Joyce J.A. (2013). Microenvironmental regulation of tumor progression and metastasis. Nat. Med..

[2] Huang Y., Kim B.Y.S., Chan C.K., Hahn S.M., Weissman I.L., Jiang W. (2018). Improving immune-vascular crosstalk for cancer immunotherapy. Nat. Rev. Immunol..

[3] Tape C.J., Ling S., Dimitriadi M., McMahon K.M., Worboys J.D., Leong H.S., Norrie I.C., Miller C.J., Poulogiannis G., Lauffenburger D.A. (2016). Oncogenic KRAS Regulates Tumor Cell Signaling via Stromal Reciprocation. Cell.

[4] Wu S.Z., Swarbrick A. (2021). Single-cell advances in stromal-leukocyte interactions in cancer. Immunol. Rev..

[5] Hanahan D., Coussens L.M. (2012). Accessories to the crime: Functions of cells recruited to the tumor microenvironment. Cancer Cell.

[6] Shi Y., Du L., Lin L., Wang Y. (2017). Tumour-associated mesenchymal stem/stromal cells: Emerging therapeutic targets. Nat. Rev. Drug Discov..

[7] Lewis C.E., Harney A.S., Pollard J.W. (2016). The Multifaceted Role of Perivascular Macrophages in Tumors. Cancer Cell.

[8] Kania A., Klein R. (2016). Mechanisms of ephrin-Eph signalling in development, physiology and disease. Nat. Rev. Mol. Cell Biol..

[9] Wilkinson D.G. (2014). Regulation of cell differentiation by Eph receptor and ephrin signaling. Cell Adh. Migr..

[10] Darling T.K., Lamb T.J. (2019). Emerging Roles for Eph Receptors and Ephrin Ligands in Immunity. Front. Immunol..

[11] Nievergall E., Lackmann M., Janes P.W. (2012). Eph-dependent cell-cell adhesion and segregation in development and cancer. Cell Mol. Life Sci..

[12] Chen J., Song W., Amato K. (2015). Eph receptor tyrosine kinases in cancer stem cells. Cytokine Growth Factor Rev..

[13] Mosch B., Reissenweber B., Neuber C., Pietzsch J. (2010). Eph receptors and ephrin ligands: Important players in angiogenesis and tumor angiogenesis. J. Oncol..

[14] Janes P.W., Vail M.E., Gan H.K., Scott A.M. (2020). Antibody Targeting of Eph Receptors in Cancer. Pharmaceuticals.

[15] Shiuan E., Chen J. (2016). Eph Receptor Tyrosine Kinases in Tumor Immunity. Cancer Res..

[16] Zhou X., Tu P., Chen X., Guo S., Wang J. (2017). Eph Receptors: Actors in Tumor Microenvironment. Crit. Rev. Oncog..

[17] Janes P.W., Vail M.E., Ernst M., Scott A.M. (2020). Eph receptors in the immune-suppressive tumor microenvironment. Cancer Res..

[18] Ogawa K., Pasqualini R., Lindberg R.A., Kain R., Freeman A.L., Pasquale E.B. (2000). The ephrin-A1 ligand and its receptor, EphA2, are expressed during tumor neovascularization. Oncogene.

[19] Brantley D.M., Cheng N., Thompson E.J., Lin Q., Brekken R.A., Thorpe P.E., Muraoka R.S., Cerretti D.P., Pozzi A., Jackson D. (2002). Soluble Eph A receptors inhibit tumor angiogenesis and progression in vivo. Oncogene.

[20] Brantley-Sieders D.M., Chen J. (2004). Eph receptor tyrosine kinases in angiogenesis: From development to disease. Angiogenesis.

[21] Janes P.W., Slape C.I., Farnsworth R.H., Atapattu L., Scott A.M., Vail M.E. (2014). EphA3 biology and cancer. Growth Factors.

[22] Vail M.E., Murone C., Tan A., Hii L., Abebe D., Janes P.W., Lee F.-T., Baer M., Palath V., Bebbington C. (2014). Targeting EphA3 Inhibits Cancer Growth by Disrupting the Tumor Stromal Microenvironment. Cancer Res..

[23] To C., Farnsworth R., Vail M., Chheang C., Gargett C., Murone C., Llerena C., Major A., Scott A., Janes P. (2014). Hypoxia-controlled EphA3 marks a human endometrium derived multipotent mesenchymal stromal cell that supports vascular growth. PLoS ONE.

[24] Dow L.E., Premsrirut P.K., Zuber J., Fellmann C., McJunkin K., Miething C., Park Y., Dickins R.A., Hannon G.J., Lowe S.W. (2012). A pipeline for the generation of shRNA transgenic mice. Nat. Protoc..

[25] Vert J.-P., Foveau N., Lajaunie C., Vandenbrouck Y. (2006). An accurate and interpretable model for siRNA efficacy prediction. BMC Bioinform..

[26] Fellmann C., Zuber J., McJunkin K., Chang K., Malone C.D., Dickins R.A., Xu Q., Hengartner M.O., Elledge S.J., Hannon G.J. (2011). Functional identification of optimized RNAi triggers using a massively parallel sensor assay. Mol. Cell.

[27] Pluta K., Luce M.J., Bao L., Agha-Mohammadi S., Reiser J. (2005). Tight control of transgene expression by lentivirus vectors containing second-generation tetracycline-responsive promoters. J. Gene Med..

[28] Premsrirut P.K., Dow L.E., Kim S.Y., Camiolo M., Malone C.D., Miething C., Scuoppo C., Zuber J., Dickins R.A., Kogan S.C. (2011). A rapid and scalable system for studying gene function in mice using conditional RNA interference. Cell.

[29] Beard C., Hochedlinger K., Plath K., Wutz A., Jaenisch R. (2006). Efficient method to generate single-copy transgenic mice by site-specific integration in embryonic stem cells. Genesis.

[30] Zambrowicz B.P., Imamoto A., Fiering S., Herzenberg L.A., Kerr W.G., Soriano P. (1997). Disruption of overlapping transcripts in the ROSA beta geo 26 gene trap strain leads to widespread expression of beta-galactosidase in mouse embryos and hematopoietic cells. Proc. Natl. Acad. Sci. USA.

[31] Takiguchi M., Dow L.E., Prier J.E., Carmichael C.L., Kile B.T., Turner S.J., Lowe S.W., Huang D.C., Dickins R.A. (2013). Variability of inducible expression across the hematopoietic system of tetracycline transactivator transgenic mice. PLoS ONE.

[32] Baker M., Robinson S.D., Lechertier T., Barber P.R., Tavora B., D’Amico G., Jones D.T., Vojnovic B., Hodivala-Dilke K. (2011). Use of the mouse aortic ring assay to study angiogenesis. Nat. Protoc..

[33] Janes P.W., Griesshaber B., Atapattu L., Nievergall E., Hii L.L., Mensinga A., Chheang C., Day B.W., Boyd A.W., Bastiaens P.I. (2011). Eph receptor function is modulated by heterooligomerization of A and B type Eph receptors. J. Cell Biol..

[34] Mills S.J., Cowin A.J., Kaur P. (2013). Pericytes, mesenchymal stem cells and the wound healing process. Cells.

[35] Dickins R.A., McJunkin K., Hernando E., Premsrirut P.K., Krizhanovsky V., Burgess D.J., Kim S.Y., Cordon-Cardo C., Zender L., Hannon G.J. (2007). Tissue-specific and reversible RNA interference in transgenic mice. Nat. Genet..

[36] Stephen L.J., Fawkes A.L., Verhoeve A., Lemke G., Brown A. (2007). A critical role for the EphA3 receptor tyrosine kinase in heart development. Dev. Biol..

[37] Alorro M.G., Pierce T.P., Eissmann M.F., Dijkstra C., Dickins R.A., Ernst M., Buchert M., Masson F. (2017). Generation of an inducible mouse model to reversibly silence Stat3. Genesis.

[38] McAllister S.S., Weinberg R.A. (2014). The tumour-induced systemic environment as a critical regulator of cancer progression and metastasis. Nat. Cell Biol..

[39] Sahai E., Astsaturov I., Cukierman E., DeNardo D.G., Egeblad M., Evans R.M., Fearon D., Greten F.R., Hingorani S.R., Hunter T. (2020). A framework for advancing our understanding of cancer-associated fibroblasts. Nat. Rev. Cancer.

[40] Wu S.Z., Roden D.L., Wang C., Holliday H., Harvey K., Cazet A.S., Murphy K.J., Pereira B., Al-Eryani G., Bartonicek N. (2020). Stromal cell diversity associated with immune evasion in human triple-negative breast cancer. EMBO J..

[41] Zhou Y., Bian S., Zhou X., Cui Y., Wang W., Wen L., Guo L., Fu W., Tang F. (2020). Single-Cell Multiomics Sequencing Reveals Prevalent Genomic Alterations in Tumor Stromal Cells of Human Colorectal Cancer. Cancer Cell.

[42] Elsafadi M., Manikandan M., Almalki S., Mahmood A., Shinwari T., Vishnubalaji R., Mobarak M., Alfayez M., Aldahmash A., Kassem M. (2020). Transgelin is a poor prognostic factor associated with advanced colorectal cancer (CRC) stage promoting tumor growth and migration in a TGFβ-dependent manner. Cell Death Dis..

[43] Davidson S., Efremova M., Riedel A., Mahata B., Pramanik J., Huuhtanen J., Kar G., Vento-Tormo R., Hagai T., Chen X. (2020). Single-Cell RNA Sequencing Reveals a Dynamic Stromal Niche That Supports Tumor Growth. Cell Rep..

[44] Wu S.Z., Al-Eryani G., Roden D.L., Junankar S., Harvey K., Andersson A., Thennavan A., Wang C., Torpy J.R., Bartonicek N. (2021). A single-cell and spatially resolved atlas of human breast cancers. Nat. Genet..

[45] Tirosh I., Izar B., Prakadan S.M., Wadsworth M.H., Treacy D., Trombetta J.J., Rotem A., Rodman C., Lian C., Murphy G. (2016). Dissecting the multicellular ecosystem of metastatic melanoma by single-cell RNA-seq. Science.

[46] Zhang L., Li Z., Skrzypczynska K.M., Fang Q., Zhang W., O’Brien S.A., He Y., Wang L., Zhang Q., Kim A. (2020). Single-Cell Analyses Inform Mechanisms of Myeloid-Targeted Therapies in Colon Cancer. Cell.

[47] Kojima Y., Acar A., Eaton E.N., Mellody K.T., Scheel C., Ben-Porath I., Onder T.T., Wang Z.C., Richardson A.L., Weinberg R.A. (2010). Autocrine TGF-beta and stromal cell-derived factor-1 (SDF-1) signaling drives the evolution of tumor-promoting mammary stromal myofibroblasts. Proc. Natl. Acad. Sci. USA.

[48] Day B.W., Stringer B.W., Al-Ejeh F., Ting M.J., Wilson J., Ensbey K.S., Jamieson P.R., Bruce Z.C., Lim Y.C., Offenhauser C. (2013). EphA3 maintains tumorigenicity and is a therapeutic target in glioblastoma multiforme. Cancer Cell.

[49] Foo S.S., Turner C.J., Adams S., Compagni A., Aubyn D., Kogata N., Lindblom P., Shani M., Zicha D., Adams R.H. (2006). Ephrin-B2 controls cell motility and adhesion during blood-vessel-wall assembly. Cell.

[50] Salvucci O., Maric D., Economopoulou M., Sakakibara S., Merlin S., Follenzi A., Tosato G. (2009). EphrinB reverse signaling contributes to endothelial and mural cell assembly into vascular structures. Blood.

[51] Motegi S.I., Ishikawa O. (2017). Mesenchymal stem cells: The roles and functions in cutaneous wound healing and tumor growth. J. Dermatol. Sci..

[52] Martin-Rendon E., Hale S.J., Ryan D., Baban D., Forde S.P., Roubelakis M., Sweeney D., Moukayed M., Harris A.L., Davies K. (2007). Transcriptional profiling of human cord blood CD133+ and cultured bone marrow mesenchymal stem cells in response to hypoxia. Stem. Cells.

[53] Öhlund D., Handly-Santana A., Biffi G., Elyada E., Almeida A.S., Ponz-Sarvise M., Corbo V., Oni T.E., Hearn S.A., Lee E.J. (2017). Distinct populations of inflammatory fibroblasts and myofibroblasts in pancreatic cancer. J. Exp. Med..

[54] Helms E., Onate M.K., Sherman M.H. (2020). Fibroblast Heterogeneity in the Pancreatic Tumor Microenvironment. Cancer Discov..

[55] Swords R.T., Greenberg P.L., Wei A.H., Durrant S., Advani A.S., Hertzberg M.S., Lewis I.D., Rivera G., Gratzinger D., Fan A.C. (2016). KB004, a first in class monoclonal antibody targeting the receptor tyrosine kinase EphA3, in patients with advanced hematologic malignancies: Results from a phase 1 study. Leuk. Res..

