# Supplementary material for: Inhibition of EphA3 Expression in Tumour Stromal Cells Suppresses Tumour Growth and Progression
Source: Cancers (Basel). 2023 Sep 20;15(18):4646. doi: 10.3390/cancers15184646 (PMC10527215; doi:10.3390/cancers15184646)
Supplement: Supplementary file 1 [file cancers-15-04646-s001.zip › cancers-2567227-supplementary.pdf]

Supplementary data

Inhibition of EphA3 expression in tumour stromal cells suppresses tumour growth and progression

Mary E. Vail [1], Rae H. Farnsworth [2,†], Linda Hii [2], Stacey Allen [1], Sakshi Arora [1], Robin L. Anderson [1], Ross A. Dickins [3], Akira Orimo [4], Sunny Z. Wu [5,‡], Alexander Swarbrick [5], Andrew M. Scott [1,2] and Peter W. Janes [1,2,*]

[1] Olivia Newton-John Cancer Research Institute and School of Cancer Medicine, La Trobe University, Heidelberg, VIC 3084, Australia
[2] Biomedicine Discovery Institute and Department of Biochemistry and Molecular Biology, Monash University, Clayton, VIC 3800, Australia; rae.farnsworth@petermac.org (R.H.F.)
[3] Australian Centre for Blood Diseases, Monash University, Melbourne, VIC 3004, Australia;
[4] Department of Pathology and Oncology, School of Medicine, Juntendo University, Tokyo 113-8421, Japan
[5] Garvan Institute of Medical Research and School of Clinical Medicine, University of NSW, Darlinghurst, NSW 2010, Australia
* Correspondence: peter.janes@onjcri.org.au
† Current address: Peter MacCallum Cancer Centre, Melbourne, VIC 3052, Australia.
‡ Current address: Genentech, South San Francisco, CA 94080, USA.

Table S1. EphA3 shRNA sequences

EphA3 190	TGCTGTTGACAGTGAGCGCTCCAACGAAGTTAATCTACTATAGTGAAGCCACAGATGTATAGTAGATTAACTTCGTTGGAATGCCTACTGCCTCGGA
EphA3 193	TGCTGTTGACAGTGAGCGAAACGAAGTTAATCTACTAGATTAGTGAAGCCACAGATGTAATCTAGTAGATTAACTTCGTTGTGCCTACTGCCTCGGA
EphA3 1182	TGCTGTTGACAGTGAGCGCTACCTTCAACATCATATGTAATAGTGAAGCCACAGATGTATTACATATGATGTTGAAGGTAATGCCTACTGCCTCGGA
EphA3 3023	TGCTGTTGACAGTGAGCGCAAGCTCTAGAAACACAATCTATAGTGAAGCCACAGATGTATAGATTGTGTTTCTAGAGCTTTTGCCTACTGCCTCGGA
EphA3 3466	TGCTGTTGACAGTGAGCGCCAGGTATTTTGTTTCTTTTAATAGTGAAGCCACAGATGTATTAAAAGAAACAAAATACCTGTTGCCTACTGCCTCGGA

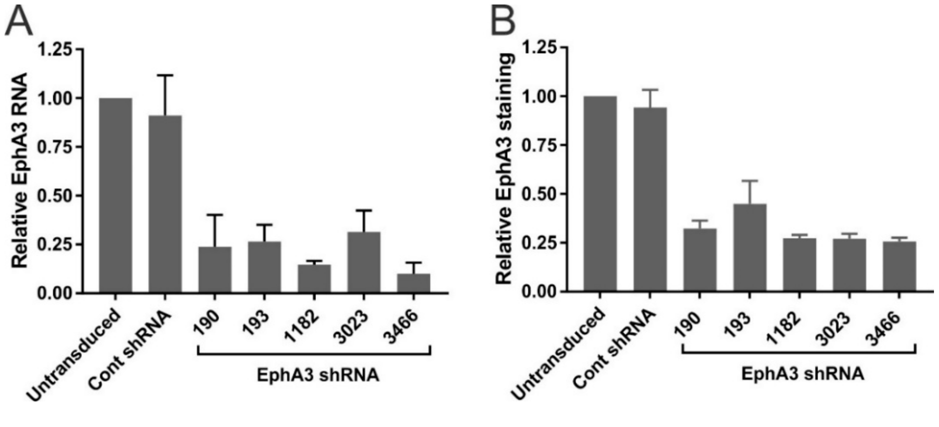

Figure S1. Screen for EphA3 shRNA knock-down in mouse embryonic fibroblasts.
MEFs transduced with control (Cont) shRNA or EphA3 shRNA were analysed for EphA3 mRNA level by qRT-PCR, normalised to housekeeping gene tubulin β4 (A), and for cell surface EphA3 protein expression by flow cytometry (B). Graphs show mean expression relative to untransduced MEFs (+/- SD, n=2).

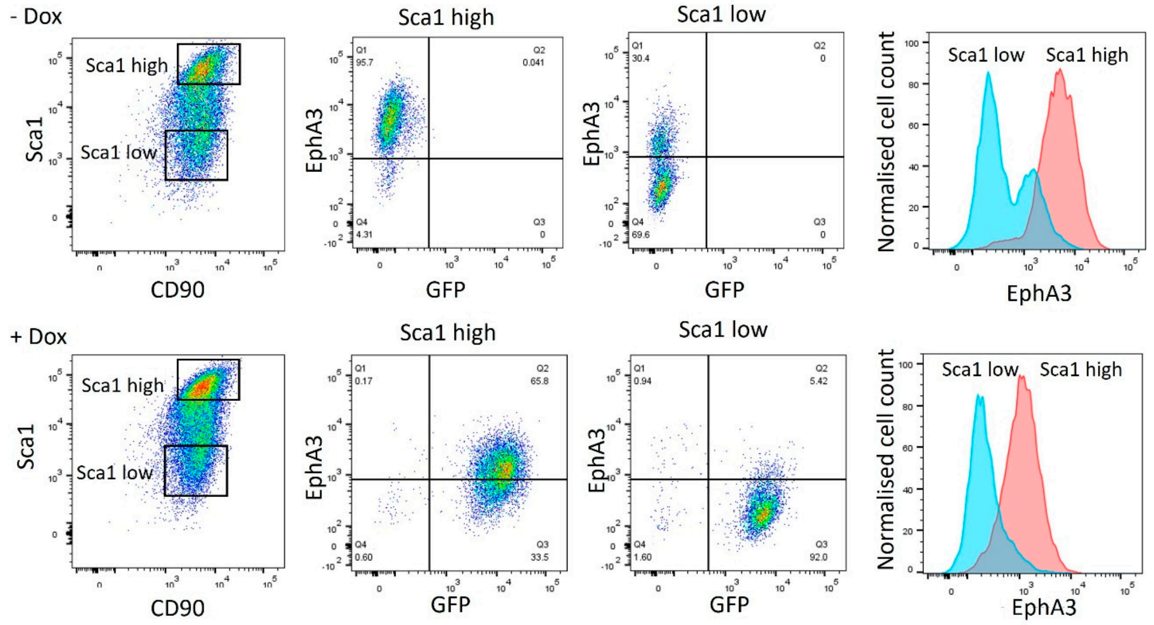

Figure S2. EphA3 expression in aortic cell cultures distinguished by Sca1 expression.
Additional data from the experiment in Figure 2 comparing EphA3 expression in cultured aortic cells with low versus high Sca1 expression, with or without EphA3 knockdown by Doxycycline (Dox)-induced shRNA expression (marked by GFP).

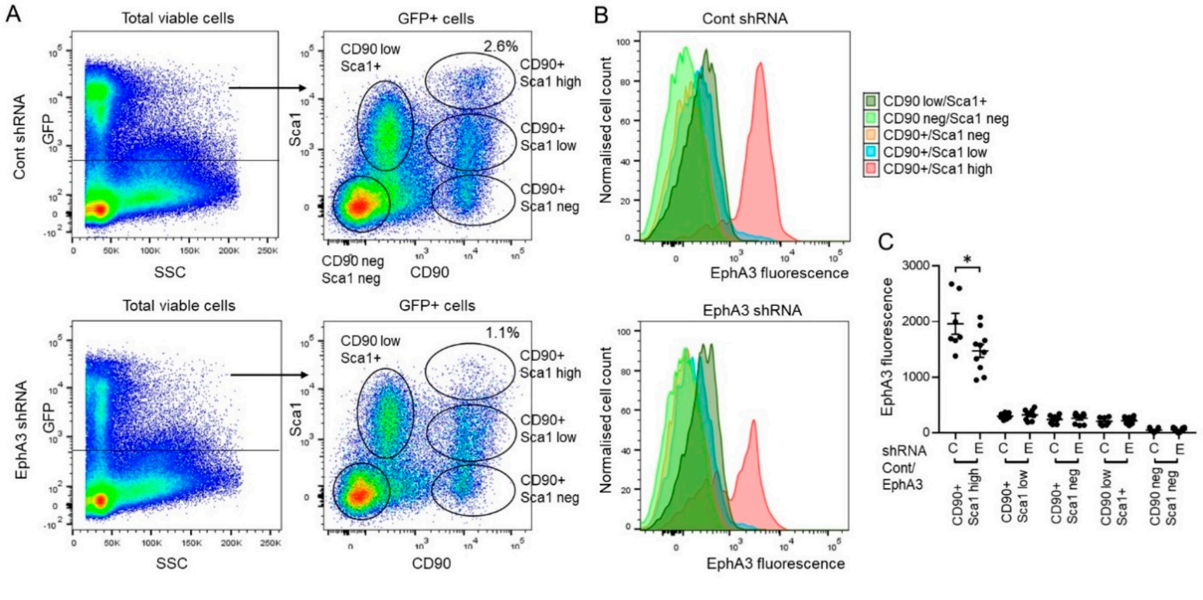

Figure S3. EphA3 expression in LLC tumour cell isolates distinguished by expression of CD90/Sca1.
Additional data from the experiment in Figure 5A comparing EphA3 expression in LLC tumour cell subpopulations from control (Cont) or EphA3 shRNA mice, distinguished by differing levels of MSC markers Sca1 and CD90. Representative flow cytometry plots show distinct cell populations (A), and histograms show associated EphA3 expression in these cell subgroups (B). C. Graph showing EphA3 expression in tumour cell populations from individual mice, with mean and SEM. (C, control shRNA; E, EphA3 shRNA); *p<0.05.

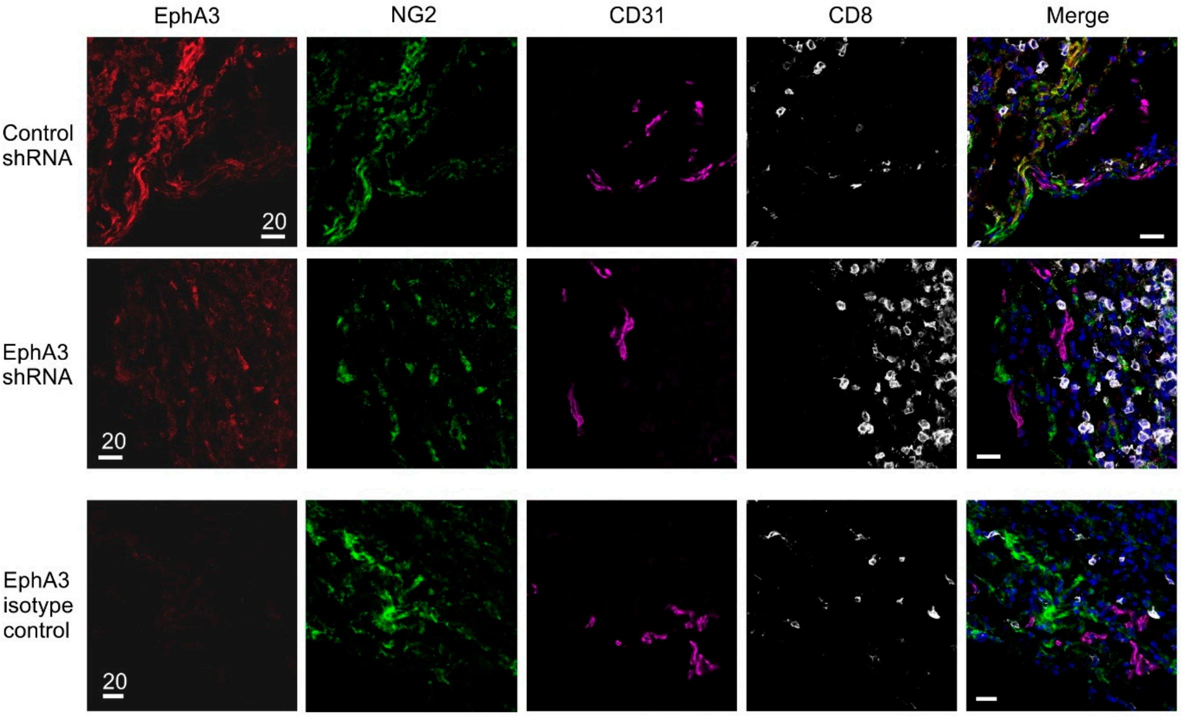

Figure S4. Immunofluorescence microscopy of LLC tumours from control and EphA3 shRNA mice.
Tissue sections were stained for EphA3, CD31+ endothelial cells, CD8+ T cells, NG2 (perivascular fibroblast-like cells) and DAPI (nuclei, blue). Yellow indicates overlap of EphA3 (red) and NG2 (green) staining. Bottom row – tumour from control mouse stained with anti-EphA3 antibody isotype control. Scale bars 20 μm.

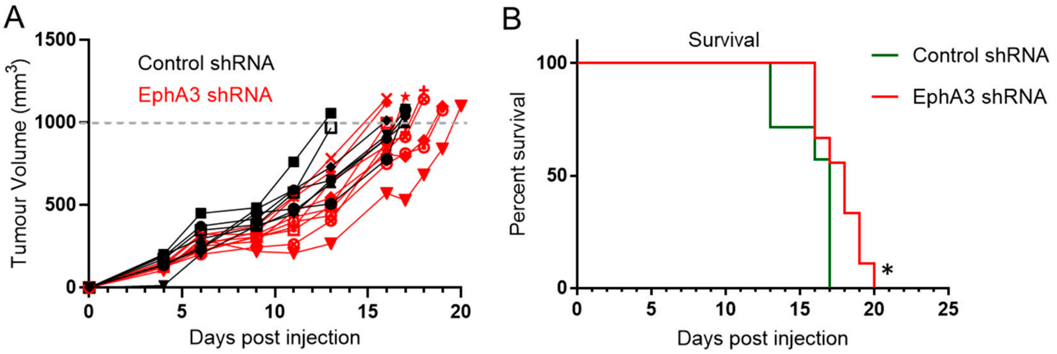

Figure S5. Tumour growth and survival of shRNA mice bearing LLC tumours.
Tumour growth (A) and associated survival analysis (B) of control and EphA3 shRNA mice after subcutaneous injection with LLC tumour cells. Time to reach ethical endpoint was significantly increased in EphA3 shRNA mice (*p<0.05, Mantel-Cox test).

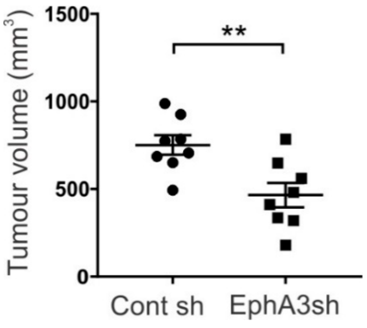

Figure S6. Effect of EphA3 knockdown on growth of B16F10 melanoma tumours.
Graph shows volumes of subcutaneous B16F10 tumours (day 12) grown in control and EphA3 shRNA mice fed doxycycline to induce shRNA expression. ** p<0.01.

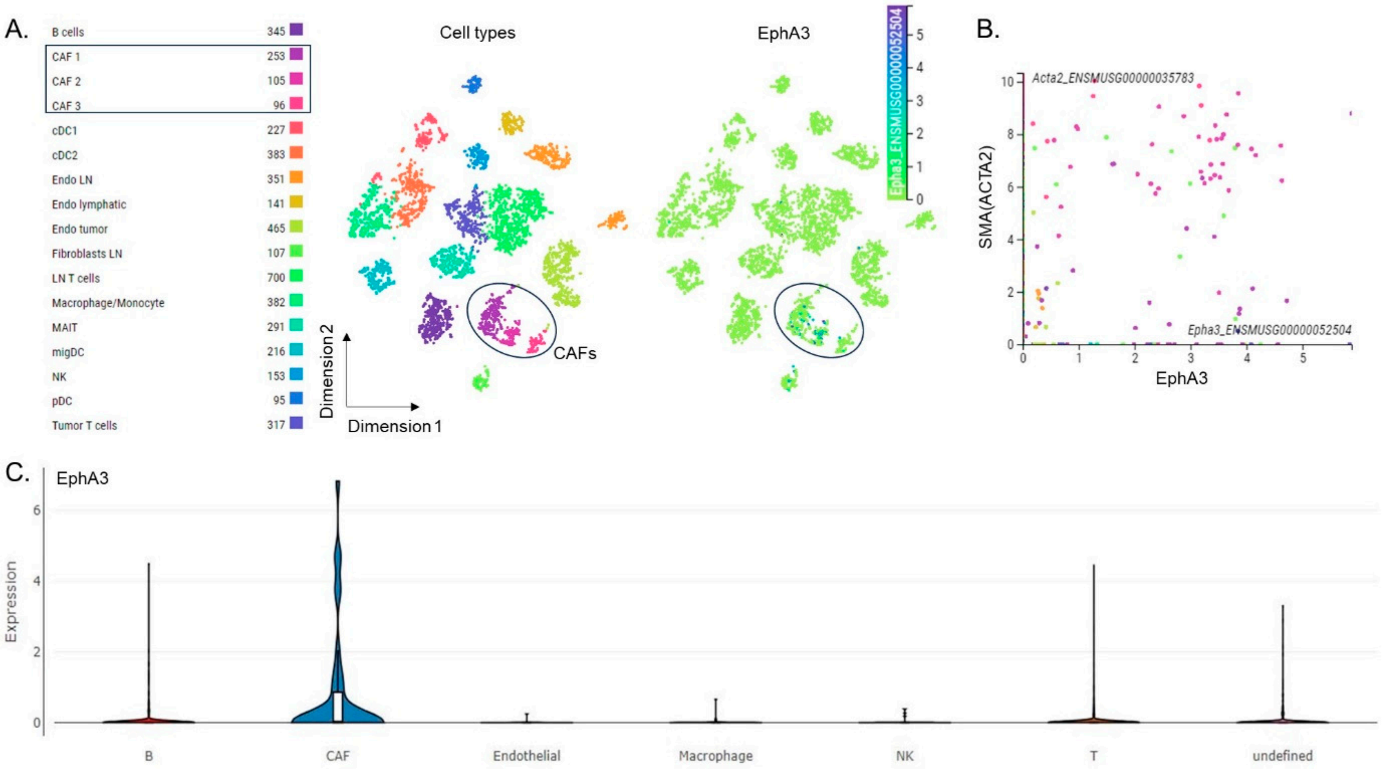

Figure S7. Analysis of single cell RNA sequencing data of mouse and human melanomas shows EphA3 in CAF subtypes.
A,B. EphA3 expression in the TME of mouse B16F10 melanoma, from Davidson et al, reference 43, https://melanoma.cellgeni.sanger.ac.uk (accessed on 7/9/2023).A. t-SNE plot of TME cell types identified by single cell sequencing, with EphA3 expression marking CAFs (right panel). B. Co-expression analysis of EphA3 with smooth muscle actin (SMA/ACTA2) in CAFs.
C. EphA3 expression in the TME of 19 human melanomas from Tirosh et al, reference 45, https://singlecell.broadinstitute.org/single_cell (accessed on 7/9/2023). Violin plots show EphA3 expression particularly in CAFs, among cell subtypes identified by single cell sequencing.
